# EcoDrug PLUS: an advanced database for drug target conservation analysis and environmental risk assessment

**DOI:** 10.1093/nar/gkaf1251

**Published:** 2025-11-22

**Authors:** Ashenafi Legehar, Siobhán Monaghan, Mael Briand, Akseli Niemelä, Leo Ghemtio, Ziaurrehman Tanoli, A Ross Brown, Charles R Tyler, Henri Xhaard

**Affiliations:** Division of Pharmaceutical Chemistry and Technology, Faculty of Pharmacy, University of Helsinki, Viikinkaari 5 E, Helsinki 00790, Finland; Biosciences, Faculty of Health and Life Sciences, Geoffrey Pope Building, University of Exeter, Stocker Road, ExeterEX4 4QD, United Kingdom; Division of Pharmaceutical Chemistry and Technology, Faculty of Pharmacy, University of Helsinki, Viikinkaari 5 E, Helsinki 00790, Finland; Division of Pharmaceutical Chemistry and Technology, Faculty of Pharmacy, University of Helsinki, Viikinkaari 5 E, Helsinki 00790, Finland; Division of Pharmaceutical Chemistry and Technology, Faculty of Pharmacy, University of Helsinki, Viikinkaari 5 E, Helsinki 00790, Finland; Institute for Molecular Medicine Finland, University of Helsinki, Helsinki00290, Finland; Biosciences, Faculty of Health and Life Sciences, Geoffrey Pope Building, University of Exeter, Stocker Road, ExeterEX4 4QD, United Kingdom; Biosciences, Faculty of Health and Life Sciences, Geoffrey Pope Building, University of Exeter, Stocker Road, ExeterEX4 4QD, United Kingdom; Division of Pharmaceutical Chemistry and Technology, Faculty of Pharmacy, University of Helsinki, Viikinkaari 5 E, Helsinki 00790, Finland

## Abstract

EcoDrug PLUS (EcoDrug+; https://ecodrugplus.helsinki.fi/) is a freely available and publicly accessible database, established to facilitate environmental risk assessment of pharmaceuticals and other bioactive substances, including veterinary medicines and pesticides. EcoDrug+ advancements on the original ECODrug database include a more extensive and intuitive graphical user interface for investigating the potential for chemicals to interact with protein targets based on their conservation with human drug, veterinary, and pesticide targets across 180 phylogenetically diverse wildlife taxa. EcoDrug+ integrates genomic and chemoinformatic data from open-access sources for ~7200 pharmaceuticals, 34 000 agrochemicals, 61 000 human metabolites, and 5800 other bioactive chemicals. Advanced search capabilities of EcoDrug+ include the ability to interrogate the database via text queries, chemical structure drawings, target protein sequence BLAST, and/or specified mechanisms of action. Chemical compound data are organized into clusters to facilitate the exploration of similar groups using interactive knowledge graphs. The integration of effects-based knowledge informs on appropriate endpoints and susceptible species for the testing of drugs (and other bioactive chemicals). Georeferenced measured environmental concentrations (for *n* = 266 chemicals) furthermore provide relevant exposure data for testing and environmental risk analysis.

## Graphical Abstract

**Graphical Abstract:**
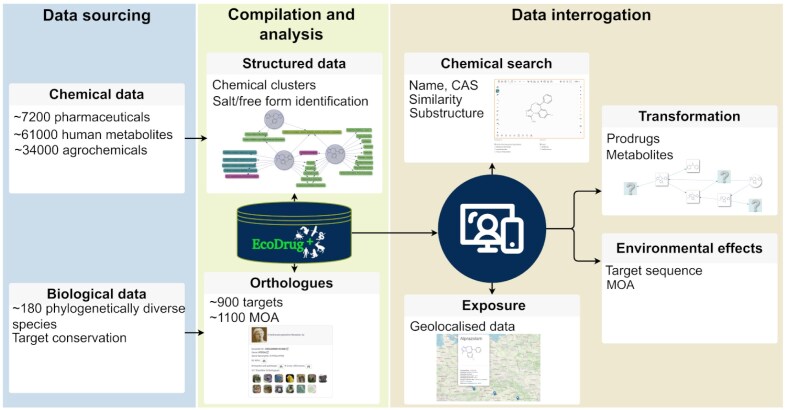

## Introduction

The widespread detection of pharmaceuticals, and other bioactive chemicals, including veterinary medicines, biocides, and pesticides in the environment has raised significant concerns regarding their potential to cause harm to both wildlife and human health [1]. Well documented examples include disruption in sexual development and function in fish from exposure to natural and synthetic hormones, antibiotic resistance in environmental bacteria transferred to human and animal pathogens, due to the use (and overuse) of antibiotics [2], and secondary poisoning, resulting in renal failure, in *Gyp* vultures in Asia after they fed on dead cattle treated with the non-steroidal anti-inflammatory drug, diclofenac. In the case of the vultures, populations were reported in 2004 to have declined by up to 95% [3]. Prominent population-level environmental impacts from other bioactive chemicals include widespread reproductive failure in marine mollusc populations in northern Europe and alligators in Florida, USA, which were attributed to tributyltin antifouling paint and organochlorine pesticides in the late 1980s and early 1990s, respectively [4, 5]. While these retrospective pathological assessments are highly informative for chemical regulation, prospective assessment tools are really needed to predict and prevent adverse effects for chronic (long-term, low-level) exposures to pharmaceuticals (and other bioactive chemicals), their excreted metabolites, transformation products, and their mixtures [6].

Active pharmaceutical ingredients (APIs) used in human and veterinary medicines are designed to inhibit or activate protein targets at submicromolar concentrations, and this can also be the case for agrochemicals and biocides. This, together with the fact that wildlife species often have highly similar (orthologous) proteins for drug, as well as for many agrochemical, targets (and off-targets) can render wildlife susceptible to exposure effects [7]. According to BLAST sequence analysis, e.g. zebrafish (used widely as a model test species) have orthologues to 86% of established drug targets in humans (*n* = 1318), [8, 9]. Furthermore, amino acid sequence identities as low as 40%–60% compared with human drug targets have been shown to confer targets for drug activation (*in vitro*) in various animal test species [10, 11], with some of these activations translating also to conserved functional (pharmacological) effects [12, 13]. Homologous proteins encoded by alternative genes in wildlife (compared to humans) have also been shown to elicit comparable pharmacological functions [14]. An example of this is the barnacle octopamine receptor that is homologous to human adrenoceptors and is activated by dexmedetomidine (the ligand for human adrenoreceptors) and produce a similar functional response [15].

Open-access tools and systems for integrating and interrogating information on drugs and other bioactive compounds are much needed for advancing their environmental risk assessment. ECODrug [16], a pioneering database, was initially developed to address these needs by providing information on drug target conservation across phylogenetically diverse environmental species. Here, we introduce a significantly more advanced and user-friendly environmental risk screening tool EcoDrug+, expanding the chemical space to include veterinary drugs, biocides and agrochemicals and combining an intuitive graphical user interface and extensive relational database (https://ecodrugplus.helsinki.fi).

## Database contents

The computational tools used are presented as Supplementary Table S1.

### Database overview and navigation

EcoDrug+ is an object-relational database developed using PostgreSQL and is hosted on cloud services provided by CSC-IT Center for Science (see Supplementary Table S1 and Supplementary Fig. S1), allowing user access at https://ecodrugplus.helsinki.fi/ from any device.

EcoDrug+ navigation is structured around four main menus: ‘Chemical’, ‘Transformation’, ‘Biochemical action’, and ‘Exposure’. Each menu offers options to browse, search, and explore the database. Searches can be also conducted from the landing page using text input, chemical structure, multiselection features for targets and mechanisms of action, or by target similarity based on a sequence of user interest. Across the webserver, database entries can be retrieved by name, by identifiers from other databases (e.g. ChEMBL ID), by chemical representations (InChi, Smiles, or InChiKey), or by drawing chemical structures. Searches are flexible, differentiating exact matches, similarity, or substructure matches within pharmaceuticals and bioactive chemicals. Target conservation can be searched using gene name, gene symbol, or Ensembl ID.

### Target conservation

EcoDrug+ stores and uses gene conservation from the orthology predictions made by Ensembl. Through the database, conservation is visually indicated by clickable species icons, representing 12–16 groups of species, that are shaded when no representative is present. Target conservation can be searched from a species perspective from the landing page (16 phylogenetic group icons), from a compound perspective from the compound pages, and from a target perspective using the ‘Biochemical action’ menu. There, ‘Targets’ tables are organized according to the ChEMBL six-level classification [17]. The ‘Biochemical action/Mechanism of action’ menu leads to the Target cards pages, where cards are boxes labelled by a gene Ensembl ID that contains a description, a mechanism of action, orthologues, and 12 phylogenetic group pictograms. The option to directly make a sequence-based search across human targets and across selected genomes through a customizable BLAST search is also provided (see below).

Ensembl annotates orthologues based on whole genomic alignments. For conserved genes, Ensembl provides gene-specific multiple amino acid sequence alignments, often in the hundreds of sequences, which should give credence to the reliability of these multiple sequence alignments. These alignments can in turn be used to calculate protein conservation, measured as a percent protein sequence identity. Ensembl uses a consensus of five reconstruction methods to automatically calculate phylogenetic trees. The Ensembl orthology annotations appear highly robust and of excellent quality, even under visual scrutiny (see e.g. [18]) and should be superior in comparison to the more simplistic methods that are based on clustering *e*-values from BLAST. Using Ensembl annotations directly should thus offer a significant improvement compared to the tripartite consensus system in the original ECOdrug database [16]. The strategy adopted to use Ensembl-annotated orthologues leaves out paralogues, however highly conserved paralogues could also exert a functional response and thus potentially adverse effects. To identify these, we created the option to conduct BLAST searches across 23 selected genomes of environmental relevance, retrieving all similar sequences to a user query.

### Database schema

**Figure 1.**
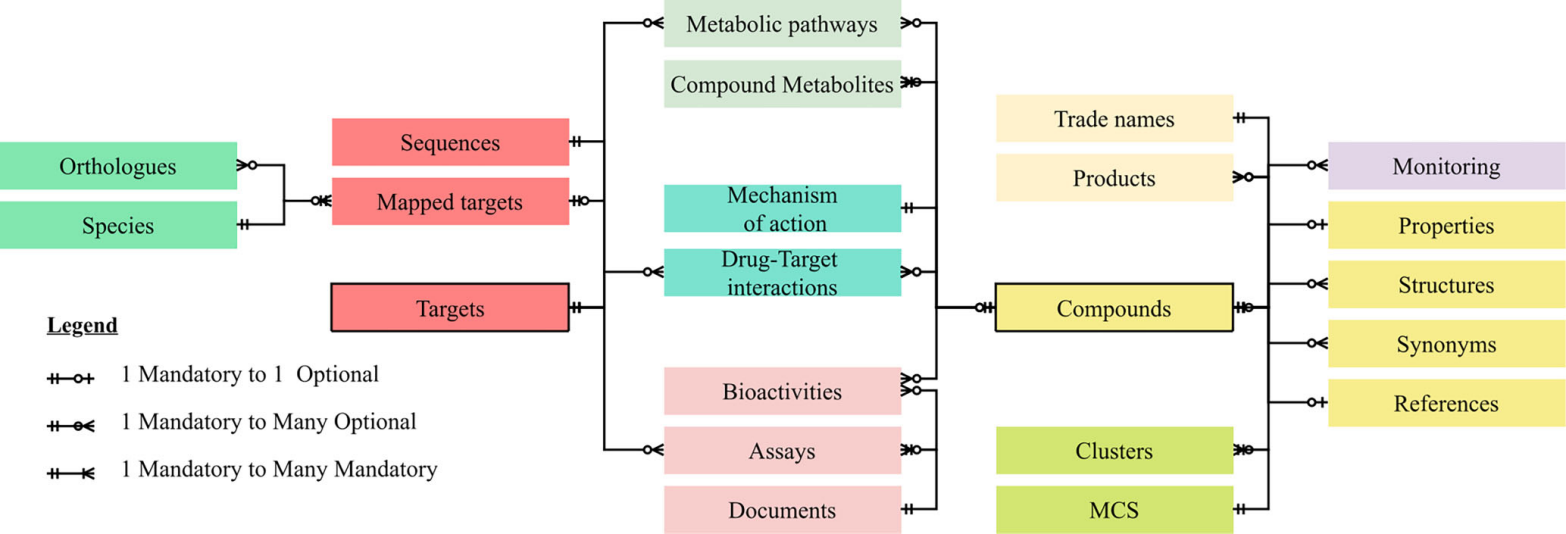
Simplified database schema for EcoDrug+ . The nine groups of data tables are indicated by the different colours.

The EcoDrug+ database is composed of nine groups of data tables (Fig. 1 and Supplementary Fig. S2), with the ‘Targets’ and ‘Compounds’ tables forming central hubs.

Targets link to Mapped targets that include identifiers for amino acid sequences, orthologues, and species in external databases, e.g. UniProt [19] and Ensembl [20]. *T*argets link to Compounds through the Drug target interaction and Biological activity tables, which contain data on experimental assay conditions and outcomes.

Compounds connect chemical compound to Properties—experimental and calculated physicochemical data; to Structures—2D compound structures; to Synonyms—References; and to Monitoring data—georeferenced measured environmental concentrations. The Compounds Table is further linked to chemical Clusters defined by their maximum common substructure (MCS); to Compound metabolites and Metabolic pathways; and to Products—information on trade names, formulation details, dosage, etc. approved by the U.S. Food and Drug Administration (FDA).

### Data sources and data integration

EcoDrug+ integrates 13 publicly accessible chemical and biological databases (Table 1), with Ensembl, ChEMBL, IUPHAR/BPS Guide to pharmacology, and NORMAN SusDat serving as the primary contributors. The data are acquired using a combination of manual and automated updates, and each data integration is documented with the specific date and database version at the time of integration. Wherever possible, data are referenced to relevant literature sources. Data preparation and processing were performed using Python scripts, SQL scripts, and Spark SQL, executed within an Apache Spark cluster environment (Supplementary Table S1 and Supplementary Fig. S1). The data for approved and investigational drugs (substance in clinical trials) were initially sourced from DrugMapper (manuscript; https://drugmapper.helsinki.fi) [21]. Each entry was assigned an EcoDrug+ identifier, and these identifiers set as primary keys, preventing data duplication. This was facilitated by using the unique ingredient identifier (UNII) extracted from the FDA Global Substance Registration System where available (Table 1).

**Table 1.** Summary of the data sources integrated within EcoDrug+

Data sources	Contents	Web URL	References
Drug@FDA	Approved drug, dosage formulation, and approval history	https://www.fda.gov/drugs/drug-approvals-and-databases/drugsfda-data-files	
Orange book	Patent and dosage formulation	https://www.fda.gov/drugs/drug-approvals-and-databases/orange-book-data-files	
FDA Global substance registration System	UNII	https://precision.fda.gov/uniisearch/archive	
ClinicalTrials	Investigational drug, maximum phase for the indication	https://aact.ctti-clinicaltrials.org/	[22]
ChEMBL v19-34	Biological activities,assay information, compound structure, mechanism of action, drug indication, metabolite, agrochemical, target and target sequence	http://ftp.ebi.ac.uk/pub/databases/chembl/ChEMBLdb/	[23]
IUPHAR/BPS	Primary target, biological activities	https://www.guidetopharmacology.org/download.jsp	[24]
PubChem	Chemical synonym, structure, and chemical information	https://ftp.ncbi.nlm.nih.gov/pubchem/	[25]
Ensembl	Orthologues gene, gene tree, gene cross reference, chromosome location	https://rest.ensembl.org/	[20]
UniProt	UniProt ID, gene symbol	https://www.uniprot.org/	[19]
Reactome	Biochemical pathway	https://reactome.org/	[26]
HUGO Gene Nomenclature Committee	Approved gene symbol	https://www.genenames.org/	[27]
NORMAN SusDat	Biocides, food additives, human metabolites, industrial chemical, plant protection products and surfactants	https://www.norman-network.com/nds/susdat/	
PHARMS-UBA	Monitoring data	https://www.umweltbundesamt.de/en/database-pharmaceuticals-in-the-environment-0	
Metabolome Database (HMDB)	Metabolites, for exogenous and endogenous compounds	https://hmdb.ca/	[28]

Orthologous genes across humans and other species were retrieved from Ensembl (version 110-112), accessed from the Ensembl REST API (Table 1). Human drug targets—as identified by ChEMBL—were used to extract the corresponding gene trees [20]. A compilation of 10 lists of compounds of potential interest, named here ‘bioactive compounds’, were collected from NORMAN SusDat database (Table 1) (drinking water chemicals; food contact chemicals; human neurotoxins; indoor environment substances; industrial chemicals; natural toxins; persistent, mobile, and toxic substances; plastic additives; smoke compounds; surfactants). Agrochemicals were collected from ChEMBL version 19 (Table 1) based on assay information compiled from Gaulton and co-workers [29]. Chemical structures were integrated in standardized formats: Standard InChI, InChIKey, canonical SMILES, and molfile. Molecular properties were computed using RDKit (Supplementary Table S1), and in addition, experimentally where available measured physicochemical properties were collected from CHEMBL. Drug metabolites pathways for 130 drugs were compiled using text mining and SQL scripting combined with manual curation (from ChEMBL and other databases). Additionally, a large dataset (~60k entries) with endogenous and exogenous metabolites was retrieved from NORMAN SusDat and mapped to Human Metabolome Database (Table 1).

## Advancements provided in EcoDrug+ versus ECOdrug

EcoDrug+ provides a significantly advanced data system compared with ECOdrug in terms of data content, including expanding the chemical space to include veterinary drugs, agrochemicals, and biocides, and with improved navigability and ease of interrogation.

### Expanded data content via direct integration and cross-referencing to external databases

In addition to the data directly integrated, EcoDrug+ is cross-referenced to an extensive series of 25 external databases (Supplementary Table S2), which can be accessed through hyperlinks listed under the ‘About-–Data sources’ submenu in the graphical user interface. Among these, compounds are cross-referenced to US-EPA CompTox [30] and Anatomical Therapeutic Chemical Classification (Supplementary information S4). Targets are cross-referenced to UniProt [19], Expression Atlas [31], WikiGene [32], GeneCards [33], and to the HGNC [27] databases. Enzymes and pathways are cross-referenced with the Reactome database for *Homo sapiens* (801 genes), *Xenopus tropicalis* (504 genes), *Bos taurus* (705 genes), and *Danio rerio* (603 genes).

The new EcoDrug+ has been significantly expanded from 1194 APIs in the original ECOdrug database to a total of 6175 unique small molecule APIs (7970 entries in total, since an API may have two entries in the database, one as a salt and the other in free form for example) (Table 2). The entries are also structured both by chemical similarity and by a category that indicates their free form/salt form/mixture status. In total, EcoDrug+ contains 111 816 entries, of which at least 79 873 correspond to unique substances (Table 2). Each entry is connected to additional information (Table 1 and Supplementary Table S2): calculated or measured physicochemical descriptors, monitoring data spatially geolocalized, biotransformation, and *in vitro* activity values.

**Table 2.** Database content, number of database entries

Dataset	Subsets	Unique substances	Salt form	Free form	Mixture	Unknown 2D structures
Small molecule APIs	Approved (FDA approved only)	3021 (1521)	1039 (716)	2341 (881)	144 (92)	77(28)
	Investigational	3154	236	3082	NA	17
Agrochemicals		29 066	578	34 210	NA	26
Bioactive chemicals		4719	935	4902	NA	0
Metabolites		44 632	3768	58 559	NA	50

NA, not available/not investigated.

EcoDrug+ presents 828 small-molecule human targets (referenced by an Ensembl gene identifier, from a pool of 920 genes) compared with 663 human targets [34] captured in EcoDrug. The 828 human target genes in EcoDrug+ could be mapped to 180 distinct species, identifying 127 219 orthologues proteins. While EcoDrug+ maps less (180) genomes in comparison ECOdrug, which provided access to over 600 eukaryotic species, the data coverage and connection in EcoDrug+ have significantly increased. The definition of orthologues is now more accurate, as this is based on phylogenetic trees reconstructed from multiple sequence alignments instead of being inferred from sequence similarities. The key species in EcoDrug+ (Supplementary Table S3) include Mammalia i.e. mammals (22 species), Pisces i.e. fish (65 sp.), Aves i.e. birds (11 sp.), and Reptilia i.e. reptiles (13 sp.). A large group of ‘other Chordates’ (35 sp.), remaining to be classified, includes Ascidia i.e. sea squirts (2 sp.) and Lamprey (1 sp.). There are also 15 invertebrate species and one fungus. Targets also have a deep level of annotation, which allows them to be separated at different levels (Supplementary Table S4): target type (e.g. receptor, enzyme, transporter, ...), family (G protein-coupled receptor, tyrosine kinase, ...), subfamily and subtypes (as annotated by ChEMBL and/or IUPHARdb). Additionally, mechanisms of action (e.g. histamine receptor agonist, histamine receptor antagonist, etc) are also annotated, comprising 1092 mechanisms (as annotated by ChEMBL, Supplementary Table S5). EcoDrug+ also maps a significant amount of *in vitro* data that will be used at later stages to improve the predictions of environmental effects (Supplementary Table S6).

**Figure 2.**
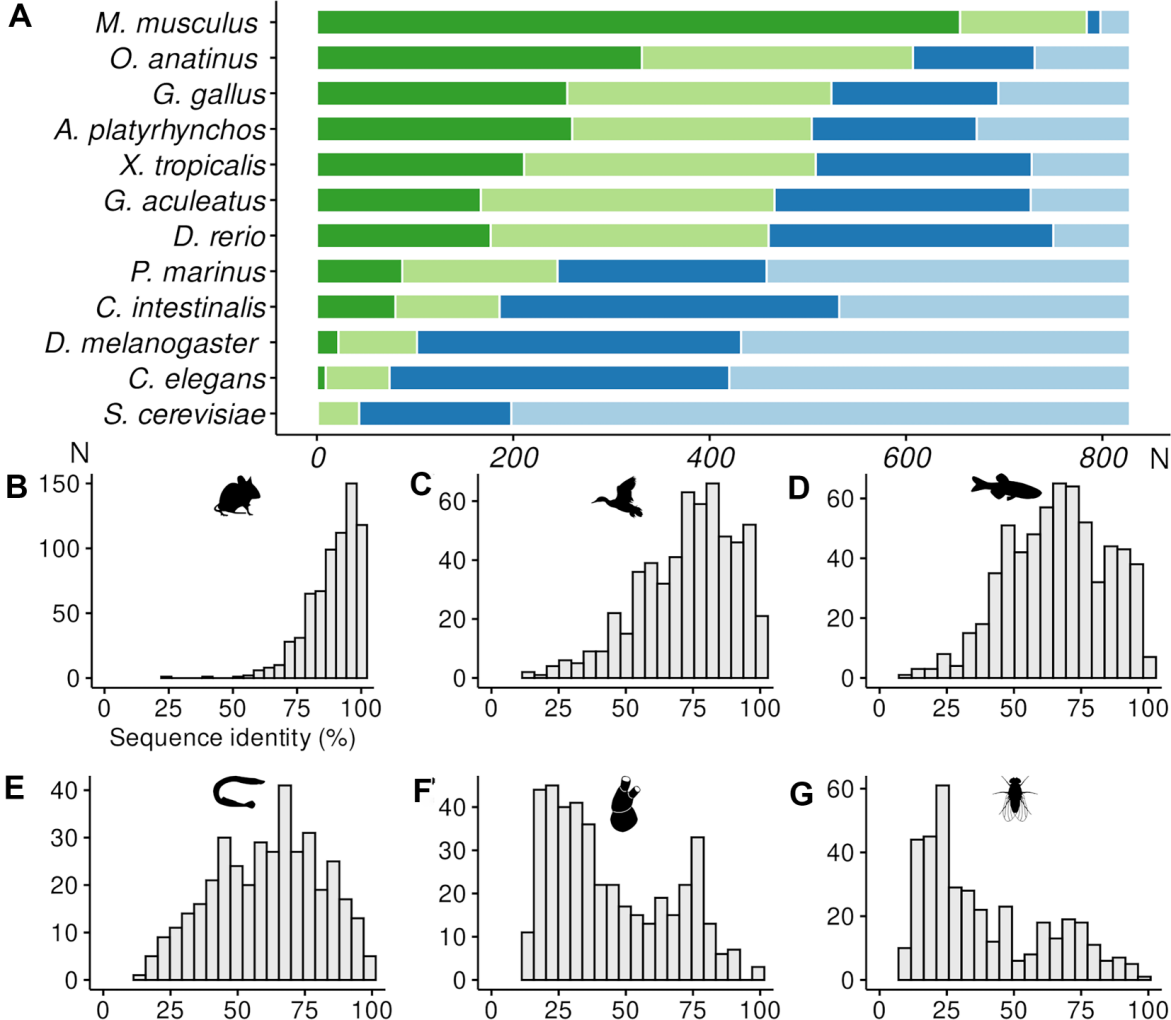
Conservation relative to 828 drug target human genes for selected species. (**A**) Predicted orthologues by Ensembl: dark green, above 80% sequence identity; light green, 80%–60% sequence identity; dark blue, below 60% sequence identity. Light blue, no predicted orthologue. (**B**–**G**) Histogram, gene counts by percent sequence identity to human genes for six selected species. (B) *Mus musculus*; (C) *Anas platyrhynchos*; (D) *Danio rerio*; (E) *Petromyzon marinus*; (F) *Ciona intestinalis*; (G) *D. melanogaster*. Silhouette images for species were downloaded from PHYLOPIC (https://www.phylopic.org/) and are used under the Creative Common licence.

EcoDrug+ allows users to consider Targets in animal species at the gene level (the conservation of orthologues). The outputs for this in EcoDrug+ are highly consistent with the work of others [9] with around 50% of the drug target genes having orthologues conserved from human to *Caenorhabditis elegans* and *Drosophila melanogaster* (Fig. 2A). The Targets mapped to EcoDrug+ also allows differentiation based on percent sequence identity calculated from the Ensembl meta-alignments (Fig. 2B), identifying which genes code for proteins that are highly conserved across species, and thus predictive of their functional conservation.

### Improved data navigation and interrogation

**Figure 3.**
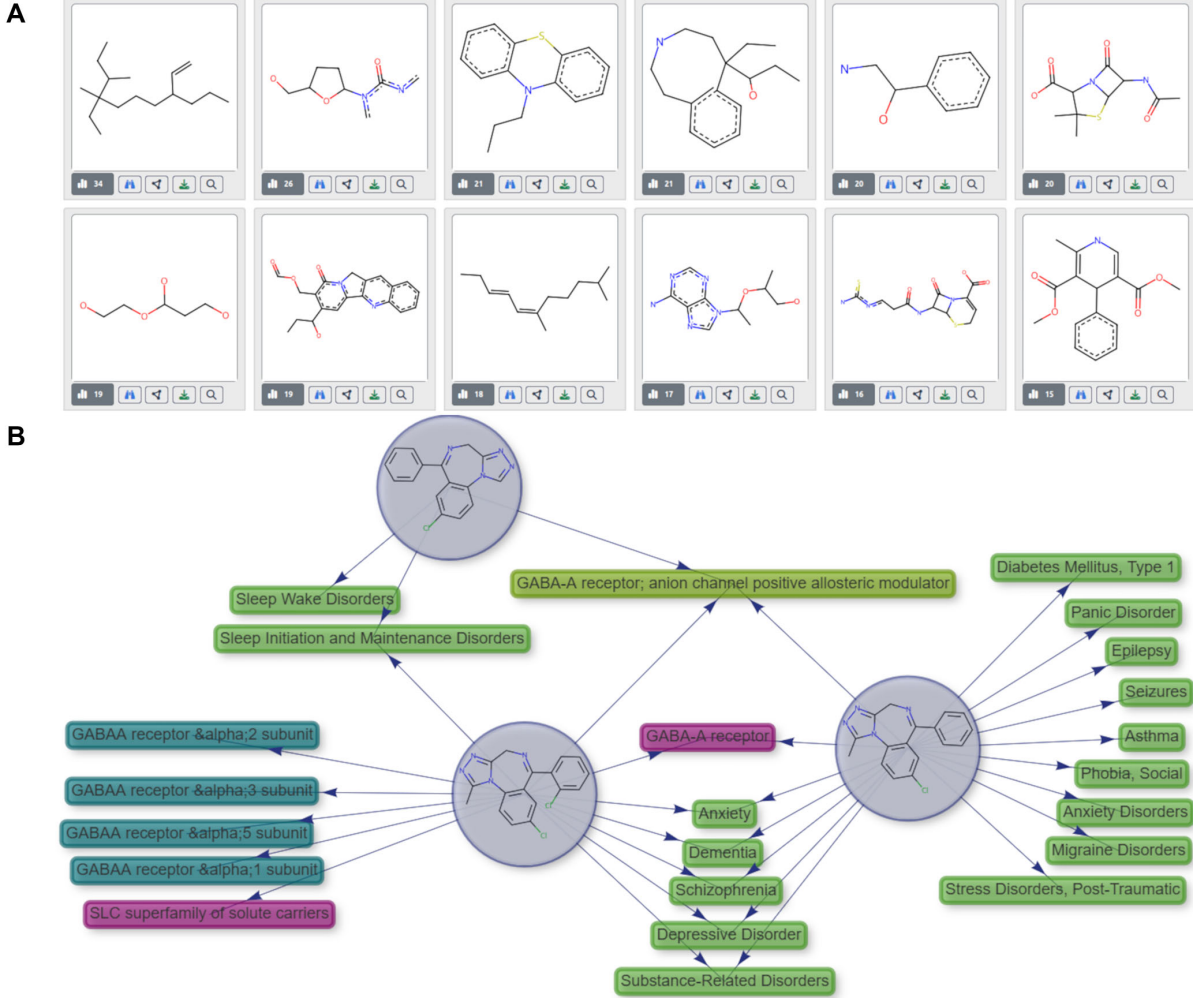
Chemical clustering: (**A**) Clusters of chemically similar entries, activated from compound page or the menu ‘Chemical/Chemical similarity’. Actionable icons: histogram, number of compounds in the cluster; binoculars, similar compounds; network, knowledge graph; arrow, download; and magnifying glass, substructure searches. (**B**) Interactive knowledge graphs allow the navigation of targets, mechanism of action, and indications for a cluster of similar compounds.

Clusters of chemically similar Compounds (Fig. 3A) can be explored from the navigational menu ‘Chemical’–‘Chemical structure’. Chemically similar pharmaceuticals and investigational compounds (~4086/7200) were divided into 526 exclusive clusters, characterized by their MCS. These clusters are also accessible through individual Compound pages.

A major advancement in EcoDrug+ compared with ECOdrug is that the Clusters and MCS tables allow users to navigate across chemically similar molecules and to build knowledge graphs connecting Compounds reflecting targets, mechanism of action, and therapeutic indications (Fig. 3B), in turn enabling inferences for compounds lacking data in these areas. To populate the Clusters and MCS tables, APIs and bioactive chemicals were clustered independently based on global chemical similarity through an unsupervised machine learning approach using RDKit (Supplementary Table S1). Additional queries can be conducted using the MCS to retrieve compounds that are similar but not included in the above clustering.

Another novel feature of EcoDrug+ is that category information (‘salt-form’, ‘free-form’, ‘mixture’, or ‘unknown’) for each entry is stored in the Compound table, and an additional table is used to store relationships (of note, the ‘free-form’ is named ‘parent’ in the database). These tables allow the user to navigate through associated data.

## Future prospects

EcoDrug+ has been built with future developments in mind. In the first instance, data inclusions can be expanded upon in terms of targets and compounds of interest. Lower order phylogenetic groups, including plants, are currently not included as they generally have low levels of human drug target conservation. Nevertheless, lower orders could be accommodated to expand EcoDrug+ to study conservation of human and non-human (e.g. pesticide) targets in these genomes using EnsemblMetazoa or EnsemblPlant.

Tracking and mapping conservation of drug targets is at the heart of EcoDrug+. The data in EcoDrug+ are currently limited to validated human drug targets, but the pool of genes encoding protein targets able to mediate physiological effects could be expanded. For example, it would be possible to add to the database potential molecular initiating events that are linked to Adverse Outcomes Pathways established in humans [35]. It is also possible that adverse effects of bioactive chemicals may be mediated through species-specific protein targets that are not considered in this version of EcoDrug+. An example here is mediation via the aryl hydrocarbon receptor in fish [36].

The EcoDrug+ database has utility to better integrate human and environmental health assessment [12]. In particular, EcoDrug+ connects to drug product data (30 232 data points). Products are associated, for example, with a certain dose/preparation marketed for a certain indication and record (e.g. adverse effects). Bioactivity tables record 388 424 data points having *in vivo* and *in vitro* pharmacological data in a broad range of species. Connecting EcoDrug+ to agrochemical and bioactive chemicals lists can benefit environmental risk screening for these chemicals. Similarly to the case for pharmaceuticals, many agrochemicals may have an identified, target-based mode of action elicited at low doses and evidenced by a significant amount of environmental data. There is also considerable potential to exploit drug-metabolite data, including the 770 entries for 130 APIs that were collected using both text mining and manually. Additionally, we collected a metabolite dataset from the NORMAN database (53 969), but these data have yet to be exploited.

Finally, EcoDrug+ can act as an extensible data repository, as an example, for environmental monitoring data. In the database established so far, georeferenced measured environmental concentrations have been compiled for 178 compounds in 16 countries. These data can be browsed in the navigation panel and each compound listed and mapped by river basins and by countries of origin.

## Conclusion

The new EcoDrug+ provides an enhanced tool for studying the potential effects of drugs and other bioactive chemicals, including agrochemicals, discharged into aquatic and terrestrial environments through a comprehensive ‘mechanism of action’-based approach. The highly structured chemical similarity grouping should allow for new annotations to be developed, associated with confidence, via chemical and target read across. The highly structured framework of the database also allows for the study of effects of drugs and other bioactive chemicals well beyond their target conservation.

## Supplementary Material

gkaf1251_Supplemental_File

## Data Availability

Ecodrug+ resource is publicly accessible at https://ecodrugplus.helsinki.fi. Users can download the entire database in various format including CSV files, and the full database SQL dump. EcoDrug+ offers a web-based graphical user interface for interactive exploration and chemical analysis. For extensive data retrieval, downloadable files are provided, along with programmatic access through the EcoDrug+ REST API (https://ecodrugplus.helsinki.fi/WebResources), which return data in responses in JSON format.
