# Supplementary material for: EcoDrug PLUS: an advanced database for drug target conservation analysis and environmental risk assessment
Source: Nucleic Acids Res. 2025 Nov 22;54(D1):D1397–404. doi: 10.1093/nar/gkaf1251 (PMC12807727; doi:10.1093/nar/gkaf1251)
Supplement: gkaf1251_Supplemental_File [file gkaf1251_supplemental_file.pdf]

Supporting Information for

EcoDrug PLUS: *An advanced database for drug target conservation analyses and environmental risk assessment*

*Ashenafi Legehar[1], Siobhán Monaghan[3], Mael Briand[1], Akseli Niemelä[1], Leo Ghemtio[1], Ziaurrehman Tanoli[2], Andrew Ross Brown[3], Charles R. Tyler[3], *, and Henri Xhaard[1], **

[1] Division of Pharmaceutical Chemistry and Technology, Faculty of Pharmacy, University of Helsinki, P.O. Box 56 (Viikinkaari 5 E) FI-00014 University of Helsinki Finland.

[2] Institute for Molecular Medicine Finland, University of Helsinki, Helsinki FI-00014, Finland

[3] Biosciences, College of Life and Environmental Sciences, Geoffrey Pope Building, University of Exeter, Stocker Road, Exeter, EX4 4QD, UK.

SUPPORTING INFORMATION 1 – Computational methods for database development

The development software and tools used are listed in Table S1.

Table S1. Software and tools

	**URL**
PostgreSQL	https://www.postgresql.org/
SQLAlchemy	https://www.sqlalchemy.org/
FLASK	https://flask.palletsprojects.com/
RDKit	https://www.rdkit.org/
Apache SPARK	https://spark.apache.org/
KNIME	https://www.knime.com/
DIAMOND	https://github.com/bbuchfink/diamond
VisNetwork	https://visjs.org/
leaflet	https://rstudio.github.io/leaflet/
R	https://www.rdkit.org/
Html widgets	https://www.htmlwidgets.org/
html tools	https://rstudio.github.io/htmltools/

Cheminformatics methods used for data mining, analysis, and visualization were conducted using RDKit, the RDKit PostgreSQL cartridge, Apache SPARK (1), Python Panda's packages (2) KNIME (3) and R (4). Additionally, RDKit along with Python scripts, were used to compute molecular descriptors, generate fingerprints, and identify maximum common substructures (MCS). For Target and sequence analysis, DIAMOND was integrated for the analysis of protein sequences and for finding targets in the database within a user specified threshold. The VisNetwork (https://visjs.org/) and HTML widgets (https://www.htmlwidgets.org/) packages in R were used to create the visualizations for drug-metabolites network, clustered compounds networks (knowledge graph). Additionally, we used leaflet (https://rstudio.github.io/leaflet/), htmlwidgets, and html tools (https://rstudio.github.io/htmltools/) in R to generate environmental monitoring for the data pharmaceuticals. Leaflet is a freely available Javascript library and generates interactive maps.

The chemical clustering was performed using the Butina clustering algorithm employing Tanimoto coefficients based on Morgan fingerprints (radius 3, 2048 bits per molecule) with a similarity threshold of 0.7. Knowledge graphs are generated in advance and pre-stored for fast access.

For salt-mixture identification, the annotation was done using the ChEMBL structure curation pipeline (5) along with a custom Python script. The workflow used for salt/mixture identification utilises text mining using the reference salt lists from the American Medical Association (https://github.com/chembl/ChEMBL_Structure_Pipeline/blob/master/chembl_structure_pipeline/data/salts.smi) (https://www.ama-assn.org/system/files/2019-04/radicals-and-anions-list.pdf).

SUPPORTING INFORMATION 2 – Web development and Server Configuration

This is a brief explanation on tools used:

- Hypertext Transfer Protocol Secure (Https) is a secure communication protocol to transfer data using encryption.
- Nginx - webserver and reverse proxy, capable of handling large volumes traffic through load and balancing.
- uWSGI - an application server that perform as a bridge between the proxy server (Nginx) and the application framework (Flask)
- Flask is web application framework based on python
- SQLAlchemy – used for Object Relational mapping (ORM) for python, enabling interaction with the PostgreSQL database using python code instead of using row SQL queries in python environment.
- PostgreSQL – relational database management system (RDBMS)

Frontend - The web interface has been developed using the FLASK web application framework (https://flask.palletsprojects.com), Jinja2 (https://jinja.palletsprojects.com), bootstrap (https://getbootstrap.com) and HTML5 were used to develop the web interface. Additionally, Ketcher (https://lifescience.opensource.epam.com/ketcher/) is integrated as a chemical drawing with a chemical drawing Graphical User Interface (GUI) plugin. Furthermore, jQuery (https://jquery.com/) is used for developing an interactive user interface.

Backend - All the computations (custom BLAST searches, chemoinformatics chemical similarity searches) are run at the CSC-IT center for science supercomputing facility (https://csc.fi/en/; Finland) in the server backend. Some of the data, in particular knowledge graphs, have been pre-computed for faster processing. This includes the graphical objects (2D compounds structures, etc). These data computed from the Ensembl sequence alignments, percent sequence identity, were also pre-computed.

The server configuration is shown as Figure S1.

Figure S1. Server configuration for the EcoDrug+

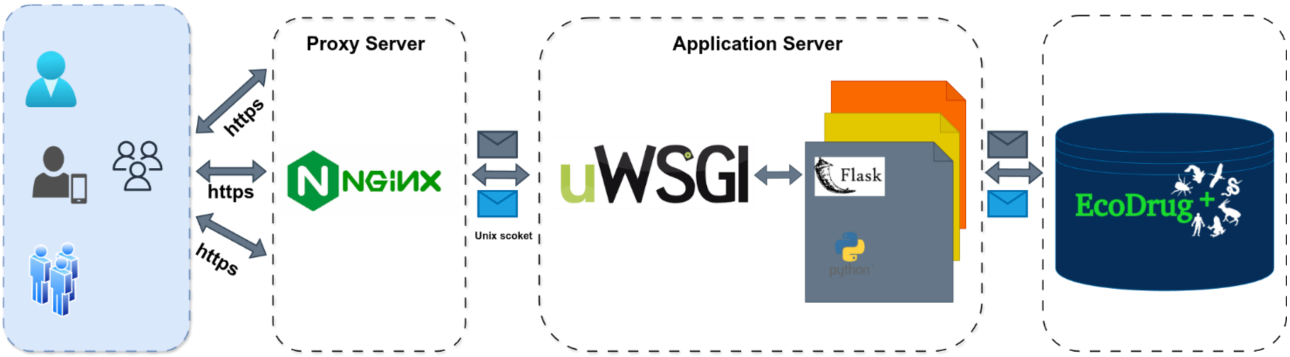

SUPPORTING INFORMATION 3 – Database Design and workflow

The EcoDrug+ full database schema is shown in Figure S2. Only the key Tables necessary for the comprehension of the databases are shown. We used the entity diagram tool from diagrams.net (https://app.diagrams.net/) for the database design.

Figure S2. EcoDrug+ full database schema. PK, Primary Key, FK, foreign key.

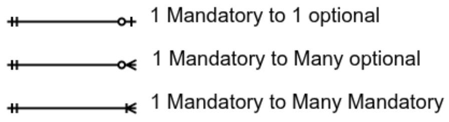

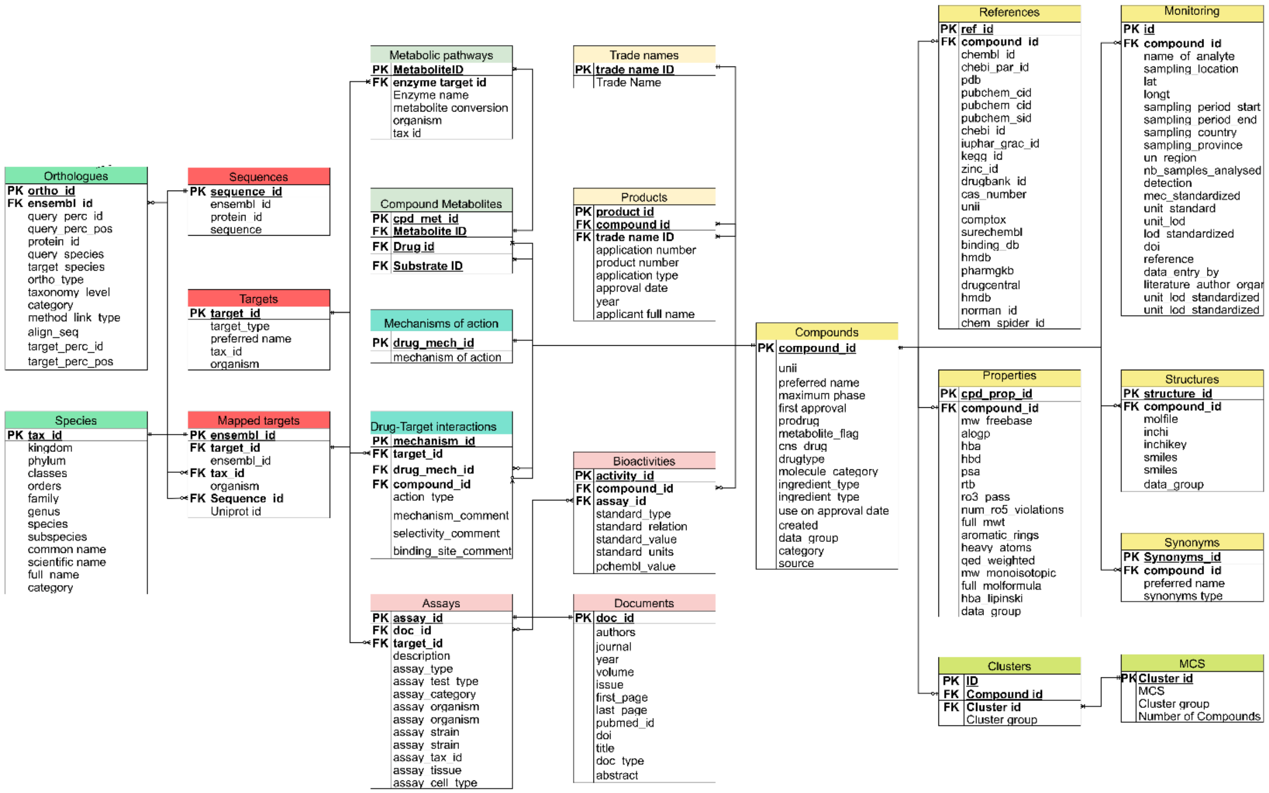

Orthologues
PK ortho_id
FK ensembl id
query perc id
query perc pos
protein id
query species
target species
ortho type
taxonomy level
category
method link type
align_seq
target_perc_id
target_perc_pos
Species
PK tax_id
kingdom
phylum
classes
orders
family
genus
species
subspecies
common name
scientific name
full name
category
Sequences
PK sequence_id
ensembl id
protein id
sequence
Targets
PK target_id
target_type
preferred name
tax id
organism
Mapped targets
PK ensembl_id
FK target_id
ensembl_id
FK tax id
organism
FK Sequence id
Uniprot id
Metabolic pathways
PK MetaboliteID
FK enzyme target id
Enzyme name
metabolite conversion
organism
tax id
Compound Metabolites
PK cpd_met_id
FK Metabolite ID
FK Drug id
FK Substrate ID
Mechanisms of action
PK drug_mech_id
mechanism of action
Drug-Target interactions
PK mechanism_id
FK target_id
FK drug_mech_id
FK compound_id
action type
mechanism_comment
selectivity_comment
binding_site_comment
Assays
PK assay_id
FK doc id
FK target_id
description
assay_type
assay test type
assay category
assay organism
assay organism
assay strain
assay strain
assay tax id
assay tissue
assay cell type
Trade names
PK trade name ID
Trade Name
Products
PK product id
FK compound id
FK trade name ID
application number
product number
application type
approval date
year
applicant full name
Bioactivities
PK activity_id
FK compound_id
FK assay_id
standard_type
standard relation
standard_value
standard units
pchembl_value
Documents
PK doc_id
authors
journal
year
volume
issue
first_page
last page
pubmed_id
doi
title
doc type
abstract
Compounds
PK compound_id
unii
preferred name
maximum phase
first approval
prodrug
metabolite_flag
cns drug
drugtype
molecule category
ingredient_type
ingredient type
use on approval date
created
data group
category
source
References
PK ref_id
FK compound id
chembl id
chebi_par_id
pdb
pubchem_cid
pubchem cid
pubchem_sid
chebi id
iuphar_grac_id
kegg id
zinc_id
drugbank id
cas_number
unii
comptox
surechembl
binding_db
hmdb
pharmgkb
drugcentral
hmdb
norman id
chem spider id
Properties
PK cpd_prop_id
FK compound_id
mw freebase
alogp
hba
hbd
psa
rtb
ro3 pass
num ro5 violations
full mwt
aromatic rings
heavy atoms
qed weighted
mw monoisotopic
full molformula
hba lipinski
data group
Clusters
PK ID
FK Compound id
FK Cluster id
Cluster group
Monitoring
PK id
FK compound id
name of analyte
sampling location
lat
longt
sampling period start
sampling period end
sampling country
sampling_province
un region
nb_samples_analysed
detection
mec_standardized
unit standard
unit_lod
lod standardized
doi
reference
data entry by
literature author orga
unit lod standardized
unit lod standardized
Structures
PK structure_id
FK compound_id
molfile
inchi
inchikey
smiles
smiles
data_group
Synonyms
PK Synonyms_id
FK compound id
preferred name
synonyms type
MCS
PK Cluster id
MCS
Cluster group
Number of Compounds

SUPPORTING INFORMATION 4 – Cross-referenced databases

The cross-referenced databases are listed in Table S2.

Table S2. Cross-referenced databases

	URL	Remark
ATC classification	https://www.who.int/tools/atc-ddd-toolkit/atc-classification	
BindingDB	https://www.bindingdb.org/rwd/bind/index.jsp	
CAS	https://www.cas.org/	
ChEMBL	https://www.ebi.ac.uk/chembl/	integrated and cross-linked
Chemical Entities of Biological Interest (ChEBI)	https://www.ebi.ac.uk/chebi/	
ChemSpider	https://www.chemspider.com/	
ClinPGx (PharmGKB)	https://www.clinpgx.org/	
DrugBank	https://go.drugbank.com/	
DrugCentral	https://drugcentral.org/	
Expression Atlas	https://www.ebi.ac.uk/gxa/home	
FDA's Global Substance Registration System	https://precision.fda.gov/uniisearch	integrated and cross-linked
GeneCards	https://www.genecards.org/	
HGNC - HUGO Gene Nomenclature Committee	https://www.genenames.org/	integrated and cross-linked
Human Metabolome Database (HMDB)	https://www.hmdb.ca/	integrated and cross-linked
IUPHAR/BPS Guide to PHARMACOLOGY	https://www.guidetopharmacology.org/	integrated and cross-linked
KEGG	https://www.genome.jp/	
NORMAN Substance Database	https://www.norman-network.com/nds/susdat/	integrated and cross-linked
PDB	https://www.rcsb.org/	
PubChem	https://pubchem.ncbi.nlm.nih.gov/	
Reactome	https://reactome.org/	
SureChEBL	https://www.surechembl.org/	
US-EPA CompTox	https://comptox.epa.gov/dashboard/	
Uniprot	https://www.uniprot.org/	
WikiGene	https://www.wikigenes.org/	
Zinc	https://zinc.docking.org/	

SUPPORTING INFORMATION 5 – Species list

Table S3. Species entries

Class	Species category	Species Entries
Amphibia	Amphibians	2
Aves	Birds	11
Fungi	Fungi	1
Invertebra	Metazoa	1
Mammalia	Afrotheria	3
Mammalia	Laurasiatheria	2
Mammalia	Marsupials and Monotremes	6
Mammalia	Other Euarchontoglires	19
Mammalia	Primates	22
Mammalia	Xenarthra	2
Pisces	Fish	65
Reptilia	Reptiles	13
Unassigned	Other chordates	35

SUPPORTING INFORMATION 6 – Target types

Table S4. Human targets (level 1 ChEMBL annotation). NA, no annotation/no data integration.

	**Approved**	**Investigational**	**Bioactive chemicals**	**Agrochemicals**	**Drug Metabolites**	**Human Metabolites**
Enzyme	1793	1308	NA	1371	4	NA
Membrane receptor	469	384	NA	599	1	NA
Transporter	183	47	NA	32	1	NA
Ion channel	153	176	NA	77	1	NA
Transcription factor	122	99	NA	261	1	NA
Unclassified protein	50	19	NA	0	0	NA
Other cytosolic protein	43	52	NA	0	0	NA
Secreted protein	23	12	NA	63	0	NA
Epigenetic regulator	20	38	NA	18	0	NA
Structural protein	9	7	NA	0	0	NA
Adhesion	5	3	NA	0	0	NA
Other nuclear protein	3	4	NA	0	0	NA
Surface antigen	2	10	NA	9	0	NA
Auxiliary transport protein	2	0	NA	0	0	NA
Other membrane protein	1	0	NA	0	0	NA

SUPPORTING INFORMATION 7 – Mechanism of Action

Table S5. The major (12) mechanisms of action, with the number of associated entries. NA, no annotation/no data integration.

Mechanism of action	Approved	Investigational	Bioactive chemicals	Agrochemicals	Drug metabolites	Human metabolites
Unknown	193	10	NA	NA	NA	NA
DNA inhibitor	73	8	NA	NA	NA	NA
Bacterial penicillin-binding protein inhibitor	69	10	NA	NA	NA	NA
Glucocorticoid receptor agonist	63	1	NA	NA	NA	NA
Histamine H1 receptor antagonist	58	5	NA	NA	NA	NA
Cyclooxygenase inhibitor	51	4	NA	NA	NA	NA
Bacterial 70S ribosome inhibitor	50	9	NA	NA	NA	NA
GABA-A receptor; anion channel positive allosteric modulator	46	1	NA	NA	NA	NA
Serotonin 2a (5-HT2a) receptor antagonist	41	11	NA	NA	NA	NA
Dopamine D2 receptor antagonist	40	9	NA	NA	NA	NA
Norepinephrine transporter inhibitor	36	5	NA	NA	NA	NA
Mu opioid receptor agonist	30	3	NA	NA	NA	NA
Muscarinic	29	7	NA	NA	NA	NA

acetylcholine receptor M3 antagonist

SUPPORTING INFORMATION 8 – Bioactivities

Table S6. Main bioactivity types, with activity relation of "=". NA, no annotations/no data integration.

Standard type	Approved	Investigational	Bioactive chemicals	Agrochemical	Drug Metabolites	Human metabolites
IC50	1238	1305	NA	8226	4	NA
Activity	0	0	NA	6396	0	NA
Inhibition	2104	1357	NA	4786	7	NA
MIC	0	0	NA	4069	0	NA
GI	0	0	NA	2824	0	NA
EC50	617	569	NA	1894	1	NA
mortality	0	0	NA	3139	0	NA
IZ	0	0	NA	2646	0	NA
Ki	816	632	NA	1087	5	NA
LC50	0	0	NA	947	0	NA
Kd	376	359	NA	150	2	NA

References

1. Zaharia M, Xin RS, Wendell P, Das T, Armbrust M, Dave A, et al. Apache spark: A unified engine for big data processing. Commun ACM. 2016 Nov 1;59(11):56–65.
2. Betancourt, R., Chen, S. (2019). pandas Library. In: Python for SAS Users. Apress, Berkeley, CA.
3. Berthold, M.R. et al. (2008). KNIME: The Konstanz Information Miner. In: Preisach, C., Burkhardt, H., Schmidt-Thieme, L., Decker, R. (eds) Data Analysis, Machine Learning and Applications. Studies in Classification, Data Analysis, and Knowledge Organization. Springer, Berlin, Heidelberg.
4. Ihaka, R., & Gentleman, R. (1996). R: A Language for Data Analysis and Graphics. Journal of Computational and Graphical Statistics, 5(3), 299–314.
5. Bento, A. P., Hersey, A., Félix, E., Landrum, G., Gaulton, A., Atkinson, F., Bellis, L. J., De Veij, M., & Leach, A. R. (2020). An open-source chemical structure curation pipeline using RDKit. Journal of cheminformatics, 12(1), 51.
